# Myocardial Infarction and Coronary Artery Disease in Menopausal Women With Type 2 Diabetes Mellitus Negatively Correlate With Total Serum Bile Acids

**DOI:** 10.3389/fendo.2021.754006

**Published:** 2021-10-05

**Authors:** Xunxun Feng, Guangyao Zhai, Jiaqi Yang, Yang Liu, Yujie Zhou, Qianyun Guo

**Affiliations:** Beijing Key Laboratory of Precision Medicine of Coronary Atherosclerotic Disease, Beijing Institute of Heart Lung and Blood Vessel Disease, Clinical Center for Coronary Heart Disease, Department of Cardiology, Beijing Anzhen Hospital, Capital Medical University, Beijing, China

**Keywords:** total serum bile acids, myocardial infarction, coronary artery disease, menopausal women, type 2 diabetes mellitus

## Abstract

**Background:**

As metabolic molecules, bile acids (BAs) not only promote the absorption of fat-soluble nutrients, but they also regulate many metabolic processes, including the homeostasis of glucose and lipids. Although total serum BA (TBA) measurement is a readily available clinical test related to coronary artery disease (CAD), myocardial infarction (MI), and type 2 diabetes mellitus (T2DM), the relationship between TBA and these pathological conditions remain unclear, and research on this topic is inconclusive.

**Methods:**

This study enrolled 20,255 menopausal women aged over 50 years, including 6,421 T2DM patients. The study population was divided into different groups according to the median TBA level in order to explore the clinical characteristics of menopausal women with different TBA levels. Spline analyses, generalized additive model (GAM) model and regression analyses based on TBA level were used to explore the relationship between TBA and different diseases independently, including CAD and MI, or in combination with T2DM.

**Results:**

Both in the general population and in the T2DM subgroup, the TBA level was significantly lower in CAD patients than in non-CAD patients. Spline analyses indicated that within normal clinical range of TBA concentration (0–10 µmol/L), the presence of CAD and MI showed similar trends in total and T2DM population. Similarly, the GAM model indicated that within the 0–10 μmol/L clinical range, the predicted probability for CAD and MI alone and in combination with T2DM was negatively correlated with TBA concentration. Multivariate regression analysis suggested that low TBA level was positively associated with the occurrence of CAD combined with T2DM (OR: 1.451; 95%CI: 1.141–1.847).

**Conclusions:**

In menopausal women, TBA may represent a valuable clinical serum marker with negative correlation for CAD and MI in patients with T2DM.

## Background

Bile acids (BAs) are endogenous metabolites synthesized from cholesterols in the liver and can be modified by intestinal microbes. Since they are important metabolic and signal transducers in the body, they may play an important role in regulating lipid and carbohydrate metabolism, as well as in shaping the composition of intestinal microbiota (1). Previous studies have emphasized potentially harmful effects of BAs in cardiovascular diseases (CVDs). For example, elevated level of BAs may have a toxic effect on the heart, and secondary BAs may promote arrhythmia (2). Recent studies have reported an important link between BAs in cholesterol metabolism and autophagic activity, suggesting that BAs may be closely related to atherosclerosis (3). Coronary artery disease (CAD) may involve general metabolic disorders. During CAD progression, phospholipid catabolism and tricarboxylic acid cycle decrease, amino acid metabolism and short-chain carnitine increase, and primary BA biosynthesis decreases. Therefore, serum metabonomics, which describes metabolic disorders, is very valuable. Differences in small molecule metabolites may reflect underlying CAD and serve as biomarkers during CAD progression (4).

From a global perspective, CVDs affect nearly 30% of patients with the type 2 diabetes mellitus (T2DM). For the past 20 years, CVDs have remained the main cause of morbidity and mortality in patients with T2DM (5, 6). In these patients, metabolomics also provides unique snapshots of biochemical changes, and may reflect unique metabolic signatures under different pathophysiological conditions. Thus, broad-spectrum metabolic changes may emphasize complex abnormalities in complications associated with elevated level of blood glucose. Hence, measuring metabolite biomarkers in blood has become a new strategy to stratify patients with T2DM complications (7). Interestingly, BAs play certain roles in glucose homeostasis, energy expenditure, and weight control, through receptor-dependent and -independent mechanisms, thereby regulating and maintaining the metabolism of lipids, glucose, and other energy sources, as well as protecting the heart from inflammation, and preventing T2DM and obesity (8–10). When BA metabolism is altered, imbalanced in lipids, glucose, and energy metabolism may lead to inflammatory metabolic diseases, including T2DM, non-alcoholic fatty liver, and CVDs (11). Moreover, serum total bile acid (TBA) is a candidate marker to predict the risk of T2DM. Previous studies have found that changes in TBA levels precede the occurrence of T2DM, supporting the potential role of TBA metabolism in T2DM pathogenesis (12). However, the specificity of TBA involvement in different populations and its exact link with the disease are still unclear.

To date, in patients with T2DM combined with CAD and myocardial infarction (MI), the correlation between TBA and disease, or the influence of TBA changes on the predicted probability of disease have not been confirmed. Besides these questions, considering potential gender differences in TBA and specific metabolic changes in menopausal women, this study aimed to explore the relationship between TBA and MI or CAD, combined or not with T2DM, based on clinical data in the population of menopausal women. The relationship between TBA and these diseases might provide helpful reference for clinicians.

## Methods

### Patient Cohort and Data Collection

**Figure 1:**
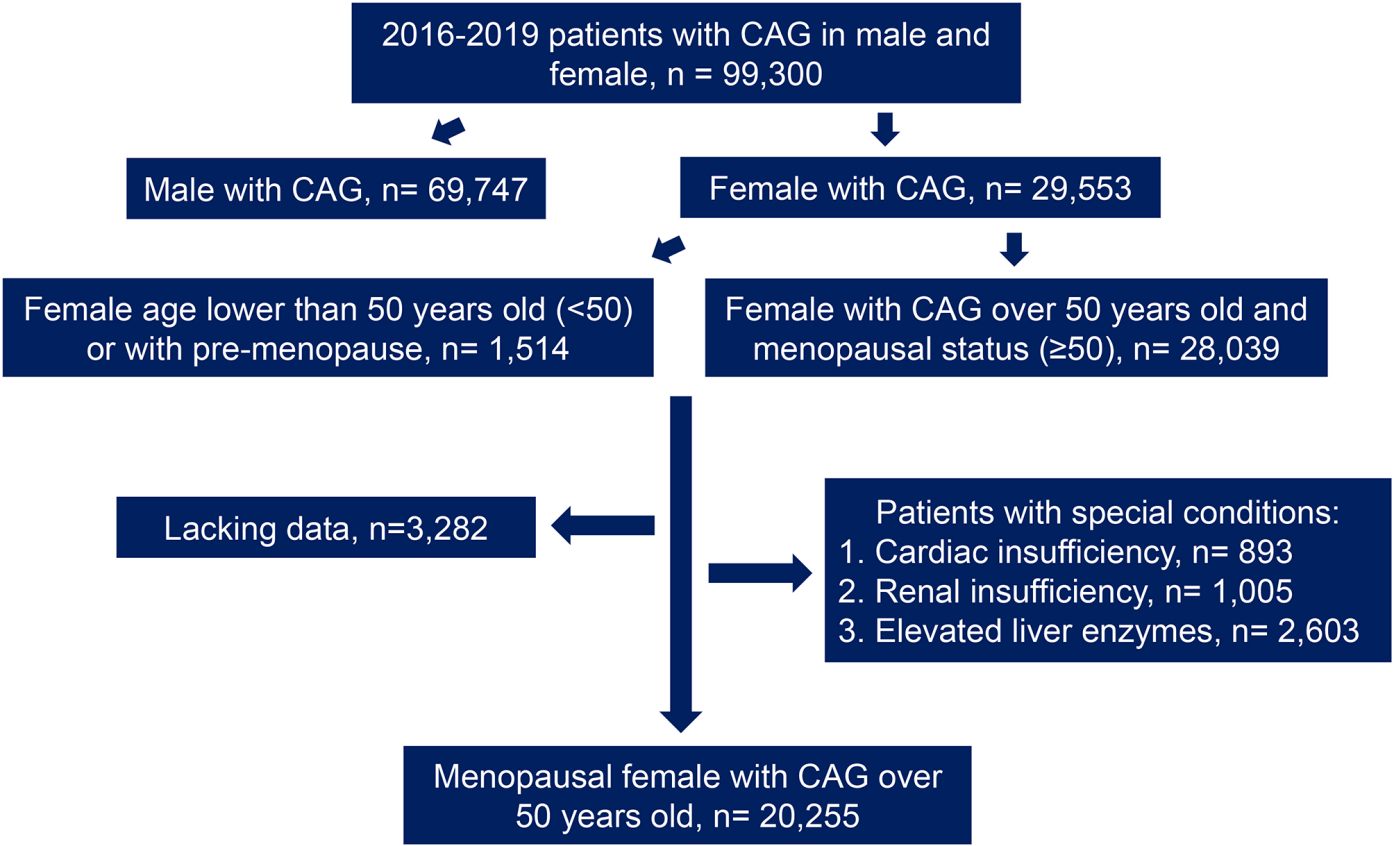
Flow chart. CAG, coronary angiography.

This study included 29,553 female patients who had undergone coronary angiography between 2016 and 2019, from the electronic database of 99,300 patients in Anzhen Hospital. According to the collected medical history and the confirmation of menstrual and childbearing history, the patients were divided into different groups as follows: menopausal women aged 50 years or more (n = 28,039) and women under 50 years or non-menopausal women (n = 1,514). Then, the data were screened through the exclusion criteria, which were as follows: cardiac insufficiency (left ventricular ejection fraction [LVEF < 50%] or a history of cardiac insufficiency, n = 893); abnormal renal function (creatinine [Cr] higher than the normal range in women from 41 to 81umol/L according to Central Laboratory of Beijing Anzhen Hospital or a history of abnormal renal function, n = 1,005); and abnormal liver function (abnormal level of alanine aminotransferase [ALT], aspartate aminotransferase [AST], gamma-glutamyl transpeptidase [γ-GGT], or a previous history of liver function abnormalities, n = 2,603). Moreover, patients with incomplete clinical data (n = 3,282) were excluded (**Figure 1**). After joint confirmation of the coronary angiography results by three experienced interventional surgeons, any coronary artery presenting with lesions narrowing more than 50% was defined as CAD. Patients with CAD were diagnosed as MI based on medical history and related examinations. For the diagnosis of MI, patients had to meet at least two of the following criteria: (1) typical chest pain; (2) elevated serum cardiac biomarkers including hypersensitive troponin I (hs-TNI), myohemoglobin and creatine kinase isoenzyme, but mainly according to hs-TNI; and (3) typical features of MI on the electrocardiogram (13).

### Laboratory Analysis

After admission, after overnight fasting, the venous blood of the participants was collected. Levels of AST, γ-GGT, albumin (ALB), triglyceride (TG), serum uric acid (SUA), total cholesterol (TC), low-density lipoprotein cholesterol (LDL-C), and high-density lipoprotein cholesterol (HDL-C) were tested using colorimetric method; Cr was detected by Jaffe’s assay; fasting blood glucose (FBG) was measured by hexose kinase method; fasting insulin and brain natriuretic peptide (BNP) were tested using chemiluminescence; ALT was tested using International Federation of Clinical Chemistry method; and total bilirubin (TBIL)/direct bilirubin (DBIL) was test using diazo method. All the abovementioned tests used in-vitro diagnostic assay kits (Roche Diagnostics, Mannheim, Germany). In addition, ion exchange high-performance liquid chromatography (HPLC) method was used to test the level of HbA1c. TBA was determined using TBA assay kit by enzyme cycle (Maccura, Chengdu, China). hs-TNI was tested by chemiluminescence using Access hs-TNI (Beckman, Minnesota, USA).

### Statistical Analysis

Data were summarized as mean standard deviation (SD), median (interquartile range), and percentage, as appropriate for continuous and categorical variables. Differences between groups were calculated using one-way analysis of variance or the Kruskal-Wallis test for continuous variables, and the chi-square test for categorical variables representing counts (percentages). Correlations between TBA and lipids characteristics were assessed using the Spearman correlation test. We evaluated the association between the level of TBA and odds ratios (ORs) of diseases with restricted cube splines (RCS), using the package of ‘RMS’ in R for the shape visualization. Besides, the generalized additive model (GAM) was applied using the package of ‘VAGM’ in R to represent the smoothing splines for disease risk prediction of changing levels of TBA (14). ORs with 95% confidence intervals (CIs) were used to present results of the logistic regression including univariate regression and multivariate regression, after adjusting the traditional influential factors, such as age, body mass index (BMI), systolic blood pressure (SBP), diastolic blood pressure (DBP), LDL-C and medical history of T2DM, hypertension (HT), hyperlipidemia, smoking, and familial CVD. All of the analyses were performed in R software (version 4.0.0).

## Results

### Baseline Clinical Characteristics

In the entire study population, the CAD group had significantly higher age, BMI, and SBP, and more frequent medical history of HT, hyperlipidemia, smoking, family CVD, and T2DMthan the non-CAD group, while DBP and alcohol history were similar between these two groups. In the T2DM subgroup, only age, SBP, medical history of hyperlipidemia, smoking history, and family CVD were significantly higher in the CAD group than in the non-CAD group. In terms of lipid composition, both in the general and in the T2DM population, the TC, HDL-C, and LDL-C levels were significantly lower in the CAD group than in the non-CAD group (P < 0.05). As for the levels of LDL-C and TC, we assumed that the lower levels in the CAD group were influenced by statins because the use of statin therapy in the CAD group was significantly more common. In contrast, the TG level was significantly higher in the CAD group than in the non-CAD group (P < 0.05). In the total population and in the T2DM subgroup, ALT and AST levels were significantly lower in the non-CAD group than in the CAD group, whereas TBIL was significantly higher in the CAD group. In terms of cardiac indicators, the level of BNP was significantly higher in the CAD group than in the non-CAD group, but there was no significant difference in TNI between the two groups. In terms of renal function, in the total population, the Cr level was significantly higher, and the estimated glomerular filtration rate (eGFR) was significantly lower in the CAD group than in the non-CAD group. Similarly, in the T2DM subgroup, the Cr level was significantly higher in the CAD group, but there was no significant difference in eGFR (P = 0.145) between the two groups. Remarkably, the TBA levels in the non-CAD group were significantly higher than those in the CAD group (median 3.70 µmol/L [IQR: 1.90-13.00 µmol/L] *vs.* 3.50 µmol/L [IQR: 1.80-11.00 µmol/L]; p < 0.001). Likewise, in the T2DM subgroup, the TBA levels in the non-CAD group were significantly higher than those in the CAD group. In terms of medications, drug use frequency in the CAD group of the total population was significantly higher than that of the non-CAD group. In the T2DM subgroup, there was no significant difference in the usage of angiotensin receptor blockers/angiotensin converting enzyme inhibitor (ARB/ACEI) between the two groups, while the frequency of usage about other drugs was significantly higher in the CAD group (**Table 1** and **Table 2**).

**Table 1:** Baseline characteristics in overall population.

Characteristics	All (n=20,255)	non-CAD (7,616)	CAD (12,639)	P value
**Age, year**	64.00 [59.00-70.00]	63.00 [58.00-68.00]	65.00 [60.00-70.00]	<0.001
**BMI, kg/m^2^**	25.51 ± 4.88	25.36 ± 3.57	25.61 ± 5.52	0.012
**SBP, mmHg**	132.51 ± 16.97	130.77 ± 16.48	133.55 ± 17.18	<0.001
**DBP, mmHg**	75.21 ± 10.39	75.40 ± 10.26	75.09 ± 10.46	0.152
**Smoking**	1,123 (5.5)	326 (4.3)	797 (6.3)	<0.001
**Drinking**	293 (1.4)	117 (1.5)	176 (1.4)	0.442
**Medical history, n (%)**				
**HT**	13,577 (67.0)	4,484 (58.9)	9,093 (71.9)	<0.001
**Hyperlipidemia**	11,439 (56.5)	4,127 (54.2)	7,312 (57.9)	<0.001
**Family CVD**	1,593 (7.9)	527 (6.9)	1,066 (8.4)	<0.001
**T2DM**	6,421 (31.7)	1,720 (22.6)	4,701 (37.2)	<0.001
**Laboratory test**				
**TC, mmol/L**	4.23 [3.63-4.95]	4.39 [3.76-5.09]	4.14 [3.57-4.86]	<0.001
**TG, mmol/L**	1.35 [0.99-1.86]	1.30 [0.96-1.79]	1.38 [1.01-1.90]	<0.001
**HDL-C, mmol/L**	1.19 [1.03-1.39]	1.23 [1.06-1.43]	1.17 [1.02-1.36]	<0.001
**LDL -C, mmol/L**	2.40 [1.89-3.04]	2.54 [1.98-3.17]	2.32 [1.84-2.92]	<0.001
**ALT, U/L**	15.00 [9.00-21.00]	15.00 [9.00-20.00]	15.00 [9.60-21.00]	<0.001
**AST, U/L**	18.00 [12.00-23.00]	18.00 [10.00-22.00]	19.00 [13.00-23.00]	<0.001
**TBIL, μmol/L**	23.00 [11.8-42.90]	36.70 [12.30-43.50]	19.90 [11.50-42.60]	<0.001
**DBIL, μmol/L**	2.04 [1.61-2.75]	2.03 [1.60-2.76]	2.04 [1.61-2.74]	0.797
**γ-GGT, U/L**	18.00 [8.13-18.60]	17.00 [5.73-25.00]	18.00 [10.00-26.00]	<0.001
**ALB, g/L**	40.80 [35.6-43.90]	40.60 [33.00-43.90]	40.90 [36.20-43.90]	0.002
**TBA, μmol/L**	3.60 [1.80-12.00]	3.70 [1.90-13.00]	3.50 [1.80-11.00]	<0.001
**hs-TNI, pg/mL**	0.01 [0.00-7.90]	0.01 [0.00-8.90]	0.01 [0.00-7.13]	0.076
**BNP, pg/mL**	55.00 [35.00-71.10]	54.70 [35.00-69.35]	55.10 [35.70-72.00]	0.025
**FBG, mmol/L**	6.18 [5.20-9.50]	5.95 [5.13-9.03]	6.37 [5.24-9.80]	<0.001
**HbA1c, %**	6.20 [5.70-7.10]	6.00 [5.60-6.60]	6.30 [5.80-7.30]	<0.001
**eGFR, mL/min/1.73m^2^**	91.64 ± 11.15	92.55 ± 11.01	91.11 ± 11.21	<0.001
**Cr, μmol/L**	44.70 ± 25.59	43.47 ± 25.73	45.45 ± 25.47	<0.001
**LVEF%**	64.79 ± 5.09	64.86 ± 5.02	64.75 ± 5.13	0.216
**Medical treatment, n (%)**				
**Aspirin**	17,344 (85.8)	5,130 (67.4)	12,214 (96.6)	<0.001
**P2Y12 inhibitors**	13,030 (64.4)	1,869 (24.5)	11,161 (88.3)	<0.001
**Statins**	16,863 (83.3)	5,309 (69.7)	11,554 (91.4)	<0.001
**Nitrate**	8,078 (40.0)	1,773 (23.3)	6,305 (49.9)	<0.001
**β-blockers**	10,673 (52.8)	3,115 (40.9)	7,558 (61.4)	<0.001
**Insulin**	1,532 (7.6)	342 (4.5)	1,190 (9.4)	<0.001
**ARB/ACEI**	3,722 (18.4)	1,177 (15.5)	2,545 (20.1)	<0.001

CAD, coronary artery disease; BMI, body mass index; SBP, systolic blood pressure; DBP, diastolic blood pressure; CVD, cardiovascular disease; T2DM, type 2 diabetes mellitus; TC, total cholesterol; TG, triglyceride; HDL-C, high-density lipoprotein cholesterol; LDL-C, low-density lipoprotein cholesterol; ALT, alanine transaminase; AST, aspartate amino transferase; TBIL, total bilirubin; DBIL, direct bilirubin; γ-GGT, gamma-glutamyl transpeptidase; ALB, albumin; TBA, total bile acid; hs-TNI, hypersensitive troponin I; BNP, brain natriuretic peptide; HbA1c, glycosylated hemoglobin A1c; eGFR, estimate glomerular filtration rate; Cr, creatinine; LVEF, left ventricular ejection fraction; β-blockers, beta-blockers; ARB, angiotensin receptor blockers; ACEI, angiotensin converting enzyme inhibitors.

**Table 2:** Baseline characteristics in T2DM subgroup.

Characteristics	All (n=6,421)	non-CAD (n=1,720)	CAD (n=4,701)	P value
**Age, year**	65.51 [61.00-70.00]	65.00 [60.00-70.00]	66.00 [61.00-71.00]	0.001
**BMI, kg/m^2^**	25.89 ± 3.43	26.06 ± 3.63	25.82 ± 3.35	0.091
**SBP, mmHg**	133.97 ± 16.86	132.48 ± 15.84	134.52 ± 17.19	0.002
**DBP, mmHg**	74.03 ± 10.05	74.40 ± 9.69	73.89 ± 10.18	0.205
**Smoking**	344 (5.4)	74 (4.3)	270 (5.7)	0.027
**Drinking**	71 (1.1)	15 (0.9)	56 (1.2)	0.343
**Medical history, n (%)**				
**HT**	5,028 (78.3)	1,322 (76.9)	3,706 (78.8)	0.096
**Hyperlipidemia**	3,807 (59.3)	1,063 (61.8)	2,744 (58.4)	0.014
**Family CVD**	513 (8.0)	116 (6.7)	397 (8.4)	0.030
**Laboratory test**				
**TC, mmol/L**	4.04 [3.48-4.78]	4.11 [3.55-4.85]	4.02 [3.46-4.75]	0.002
**TG, mmol/L**	1.40 [1.02-1.96]	1.36 [1.01-1.88]	1.42 [1.03-1.98]	0.016
**HDL-C, mmol/L**	1.63 [0.98-1.31]	1.16 [1.00-1.34]	1.12 [0.97-1.29]	<0.001
**LDL -C, mmol/L**	2.39 [1.80-2.89]	2.30 [1.85-2.98]	2.27 [1.79-2.85]	0.005
**ALT, U/L**	15.00 [10.00-21.00]	15.00 [9.00-21.00]	15.00 [10.00-22.00]	0.001
**AST, U/L**	16.62 [12.00-22.00]	17.00 [10.00-22.00]	18.00 [13.00-22.00]	0.001
**TBIL, μmol/L**	26.48 [11.20-42.70]	36.50 [11.60-43.90]	17.90 [11.10-42.30]	<0.001
**DBIL, μmol/L**	2.04 [1.60-2.73]	2.03 [1.60-2.72]	2.04 [1.60-2.73]	0.729
**γ-GGT, U/L**	19.00 [11.00-27.00]	18.00 [5.62-26.00]	19.00 [12.00-27.00]	0.003
**ALB, g/L**	40.70 [36.00-43.80]	40.60 [33.65-43.70]	40.70 [36.30-43.80]	0.264
**TBA, μmol/L**	3.60 [1.60-11.00]	3.90 [2.10-13.00]	3.50 [1.90-10.50]	<0.001
**hs-TNI, pg/mL**	0.01 [0.00-7.00]	0.01 [0.00-8.60]	0.01 [0.00-5.90]	0.222
**BNP, pg/mL**	55.45 [35.25-73.00]	54.50 [33.00-69.00]	55.80 [36.00-74.00]	0.008
**FBG, mmol/L**	8.69 [6.58-12.90]	8.36 [6.45-13.00]	8.79 [6.62-12.87]	0.150
**HbA1c, %**	7.40 [6.70-8.50]	7.20 [6.50-8.20]	7.50 [6.70-8.60]	<0.001
**eGFR, mL/min/1.73m2**	90.72 ± 11.58	91.22 ± 11.39	90.54 ± 11.65	0.152
**Cr, μmol/L**	45.84 ± 25.72	44.02 ± 26.23	46.51 ± 25.51	0.001
**LVEF, %**	64.58 ± 5.15	65.03 ± 4.90	64.43 ± 5.22	0.003
**Medical treatment, n (%)**				
**Aspirin**	5,891 (91.7)	1,344 (78.1)	4,547 (96.7)	<0.001
**P2Y12 inhibitors**	4,763 (74.2)	540 (31.4)	4,223 (89.8)	<0.001
**Statins**	5,693 (88.7)	1,386 (80.6)	4,307 (91.6)	<0.001
**Nitrate**	3,009 (46.9)	557 (32.4)	2,452 (52.2)	<0.001
**β blockers**	3,936 (61.3)	870 (50.6)	3,066 (65.2)	<0.001
**Insulin**	1,496 (23.3)	327 (19.0)	1,169 (24.9)	<0.001
**ARB/ACEI**	1,439 (22.4)	361 (21.0)	1,078 (22.9)	0.105

T2DM, type 2 diabetes mellitus; CAD, coronary artery disease; BMI, body mass index; SBP, systolic blood pressure; DBP, diastolic blood pressure; CVD, cardiovascular disease; TC, total cholesterol; TG, triglyceride; HDL-C, high-density lipoprotein cholesterol; LDL-C, low-density lipoprotein cholesterol; ALT, alanine transaminase; AST, aspartate amino transferase; TBIL, total bilirubin; DBIL, direct bilirubin; γ-GGT, gamma-glutamyl transpeptidase; ALB, albumin; TBA, total bile acid; hs-TNI, hypersensitive troponin I; BNP, brain natriuretic peptide; HbA1c, glycosylated hemoglobin A1c; eGFR, estimate glomerular filtration rate; Cr, creatinine; LVEF, left ventricular ejection fraction; β-blockers, beta-blockers; ARB, angiotensin receptor blockers; ACEI, angiotensin converting enzyme inhibitors.

### Characteristics of the Groups With Different TBA Levels

Based on the median level of TBA, three groups with gradual increase in TBA level, including low, medium, and high, were formed. In the overall population, medical history of hyperlipidemia, BNP, FBG and HbA1c level showed a gradually increasing trend mirroring the increase in TBA level in the three groups. However, medical history of familial CVD, CAD, and MI and the levels of TC, LDL-C, ALT, DBIL, and ALB showed a gradually decreasing trend. In the T2DM subgroup, medical history of HT or hyperlipidemia, and BNP level showed a gradually increasing trend, whereas medical history of family CVD, CAD, and MI and the levels of TC, DBIL, and ALB showed a gradually decreasing trend. In terms of medications, the proportions of individuals using aspirin, clopidogrel, nitrates, and beta blockers (β-blockers) in both the total and the T2DM populations declined gradually, whereas ARB/ACEI usage in the total population rose gradually (**Table 3** and **Table 4**).

**Table 3:** Characteristics of patients in different TBA levels.

Characteristics	<3.6 μmol/L (n=10,069)	3.6~10 μmol/L (n=4731)	>10 μmol/L (n=5455)	P value
**Age, year**	64.00 [59.00-69.00]	65.00 [60.00-71.00]	65.00 [60.00-70.00]	<0.001
**BMI, kg/m^2^**	25.52 ± 3.43	25.25 ± 3.41	25.61 ± 6.01	0.031
**SBP, mmHg**	132.96 ± 17.38	131.92 ± 16.49	132.44 ± 16.88	0.094
**DBP, mmHg**	75.38 ± 10.43	73.89 ± 10.10	75.59 ± 10.42	<0.001
**Smoking**	530 (5.3)	273 (5.8)	320 (5.9)	0.217
**Drinking**	106 (1.1)	57 (1.2)	130 (2.4)	<0.001
**Medical history, n (%)**				
**HT**	6,748 (67.0)	3,165 (66.9)	3,664 (67.2)	0.959
**Hyperlipidemia**	4,977 (49.4)	2,437 (51.5)	4,025 (73.8)	<0.001
**Family CVD**	895 (8.9)	353 (7.5)	345 (6.3)	<0.001
**T2DM**	3,153 (31.3)	1,588 (33.6)	1,680 (30.8)	0.006
**Laboratory test**				
**TC, mmol/L**	4.27 [3.66-4.99]	4.21 [3.61-4.95]	4.19 [3.58-4.90]	<0.001
**TG, mmol/L**	1.34 [0.99-1.84]	1.37 [1.00-1.88]	1.36 [1.00-1.88]	0.014
**HDL-C, mmol/L**	1.20 [1.04-1.40]	1.17 [1.02-1.36]	1.19 [1.03-1.39]	<0.001
**LDL -C, mmol/L**	2.42 [1.91-3.06]	2.39 [1.89-3.04]	2.36 [1.84-3.00]	<0.001
**ALT, U/L**	17.00 [13.00-23.00]	16.00 [12.00-23.00]	6.30 [5.80-7.70]	<0.001
**AST, U/L**	20.00 [17.00-24.00]	20.00 [17.00-24.00]	1.46 [0.62-4.80]	<0.001
**TBIL, μmol/L**	12.30 [10.10-15.40]	12.20 [9.80-16.20]	42.80 [40.10-45.60]	<0.001
**DBIL, μmol/L**	2.37 [1.85-3.03]	2.25 [1.72-2.92]	1.62 [1.44-1.83]	<0.001
**γ-GGT, U/L**	21.00 [16.00-28.00]	21.00 [16.00-28.00]	2.92 [2.13-4.12]	<0.001
**ALB, g/L**	42.50 [40.00-44.90]	41.40 [39.20-43.80]	22.00 [18.00-29.00]	<0.001
**TBA, μmol/L**	1.80 [1.20-2.60]	5.20 [4.20-6.70]	20.00 [15.00-28.00]	<0.001
**hs-TNI, pg/mL**	0.00 [0.00-0.01]	0.00 [0.00-0.08]	11.18 [8.44-14.40]	<0.001
**BNP, pg/mL**	42.00 [22.00-82.00]	50.00 [26.00-88.00]	58.80 [51.90-66.85]	<0.001
**FBG, mmol/L**	5.99 [5.24-8.40]	6.51 [5.39-9.76]	6.71 [4.10-14.93]	<0.001
**HbA1c, %**	6.10 [5.70-7.00]	6.20 [5.80-7.10]	6.30 [5.64-7.68]	<0.001
**eGFR, mL/min/1.73m2**	92.25 ± 11.05	91.26 ± 10.95	91.42 ± 11.28	0.001
**Cr, μmol/L**	58.18 ± 10.64	56.75 ± 15.34	9.40 ± 17.60	<0.001
**LVEF%**	65.06 ± 5.16	64.77 ± 5.15	64.14 ± 4.80	<0.001
**Medical treatment, n (%)**				
**Aspirin**	8,793 (87.3)	3,975 (84.0)	4,576 (83.9)	<0.001
**P2Y12 inhibitors**	6,865 (68.2)	3,115 (65.8)	3,050 (55.9)	<0.001
**Statins**	8,462 (84.0)	3,856 (81.5)	4,545 (83.3)	0.001
**Nitrate**	4119 (40.9)	1864 (39.4)	2095 (38.4)	0.007
**β-blockers**	5,565 (55.3)	2,533 (53.5)	2,775 (50.9)	<0.001
**Insulin**	740 (7.3)	368 (7.8)	424 (7.8)	0.518
**ARB/ACEI**	1,641 (16.3)	811 (17.1)	1,270 (23.3)	<0.001
**Disease entity, n (%)**				
**MI**	991 (9.8)	456 (9.6)	507 (9.3)	0.544
**CAD**	6,419 (63.8)	2,931 (62.0)	3,289 (60.3)	<0.001

CAD, coronary artery disease; TBA, total bile acid; BMI, body mass index; SBP, systolic blood pressure; DBP, diastolic blood pressure; CVD, cardiovascular disease; T2DM, type 2 diabetes mellitus; TC, total cholesterol; TG, triglyceride; HDL-C, high-density lipoprotein cholesterol; LDL-C, low-density lipoprotein cholesterol; ALT, alanine transaminase; AST, aspartate amino transferase; TBIL, total bilirubin; DBIL, direct bilirubin; γ-GGT, gamma-glutamyl transpeptidase; ALB, albumin; hs-TNI, hypersensitive troponin I; BNP, brain natriuretic peptide; HbA1c, glycosylated hemoglobin A1c; eGFR, estimate glomerular filtration rate; Cr, creatinine; LVEF, left ventricular ejection fraction; β-blockers, beta-blockers; ARB, angiotensin receptor blockers; ACEI, angiotensin converting enzyme inhibitors.

**Table 4:** Characteristics of patients with T2DM in different TBA levels.

Characteristics	<3.8 μmol/L (n=3,282)	3.8~10 μmol/L (n=1,459)	>10 μmol/L (n=1,680)	P value
**Age, year**	65.00 [60.00-70.00]	67.00 [62.00-72.00]	66.00 [61.00-70.00]	<0.001
**BMI, kg/m^2^**	25.77 ± 3.46	25.88 ± 3.34	25.97 ± 3.44	0.356
**SBP, mmHg**	134.70 ± 17.31	133.12 ± 16.25	133.83 ± 16.80	0.162
**DBP, mmHg**	74.32 ± 9.95	72.17 ± 9.49	74.57 ± 10.25	<0.001
**Smoking**	166 (5.1)	88 (6.0)	90 (5.4)	0.389
**Drinking**	32 (1.0)	13 (0.9)	26 (1.5)	0.127
**Medical history, n (%)**				
**HT**	2561 (78.0)	1139 (78.1)	1328 (79.0)	0.691
**Hyperlipidemia**	1689 (51.5)	820 (56.2)	1298 (77.3)	<0.001
**Family CVD**	292 (8.9)	123 (8.4)	98 (5.8)	0.001
**Laboratory test**				
**TC, mmol/L**	4.07 [3.52-4.78]	4.06 [3.50-4.84]	3.98 [3.38-4.71]	0.001
**TG, mmol/L**	1.37 [1.02-1.93]	1.46 [1.03-1.99]	1.43 [1.03-1.99]	0.018
**HDL-C, mmol/L**	1.14 [0.99-1.33]	1.11 [0.98-1.29]	1.13 [0.98-1.31]	0.016
**LDL -C, mmol/L**	2.29 [1.83-2.89]	2.29 [1.81-2.95]	2.23 [1.76-2.82]	0.001
**ALT, U/L**	17.00 [13.00-23.00]	17.00 [12.00-24.00]	7.70 [6.80-9.20]	<0.001
**AST, U/L**	19.00 [16.00-23.00]	19.00 [16.00-24.00]	1.67 [0.71-5.48]	<0.001
**TBIL, μmol/L**	11.90 [9.80-14.70]	11.54 [9.43-15.10]	42.70 [39.90-45.60]	<0.001
**DBIL, μmol/L**	2.34 [1.84-2.98]	2.23 [1.72-2.88]	1.60 [1.43-1.81]	<0.001
**γ-GGT, U/L**	22.00 [17.00-29.00]	22.00 [17.00-30.00]	2.88 [2.12-4.11]	<0.001
**ALB, g/L**	42.20 [39.70-44.80]	41.40 [39.10-43.80]	21.00 [17.00-28.00]	<0.001
**TBA, μmol/L**	2.00 [1.30-2.70]	5.20 [4.30-6.70]	21.00 [15.28-28.00]	<0.001
**hs-TNI, pg/mL**	0.00 [0.00-0.02]	0.00 [0.00-0.03]	10.99 [8.27-14.04]	<0.001
**BNP, pg/mL**	44.00 [22.00-87.00]	50.00 [28.00-93.00]	59.30 [52.20-67.85]	<0.001
**FBG, mmol/L**	8.47 [6.74-11.84]	9.27 [7.02-13.16]	8.36 [4.70-18.00]	<0.001
**HbA1c, %**	7.40 [6.70-8.50]	7.40 [6.70-8.40]	7.60 [6.60-8.80]	0.846
**eGFR, mL/min/1.73m2**	91.44 ± 11.75	90.25 ± 11.18	90.44 ± 11.61	0.017
**Cr, μmol/L**	58.70 ± 11.50	58.35 ± 14.40	9.92 ± 18.32	<0.001
**LVEF, %**	64.84 ± 5.30	64.52 ± 5.17	64.05 ± 4.71	<0.001
**Medical treatment, n (%)**				
**Aspirin**	3030 (92.3)	1334 (91.4)	1527 (90.9)	0.198
**P2Y12 inhibitors**	2556 (77.9)	1109 (76.0)	1098 (65.4)	<0.001
**Statins**	2913 (88.8)	1287 (88.2)	1493 (88.9)	0.820
**nitrate**	1570 (47.8)	675 (46.3)	764 (45.5)	0.252
**β blockers**	2050 (62.5)	907 (62.2)	979 (58.3)	0.012
**insulin**	751 (22.9)	328 (22.5)	417 (24.8)	0.218
**ARB/ACEI**	677 (20.6)	291 (19.9)	471 (28.0)	<0.001
**Disease entity, n (%)**				
**MI**	419 (12.8)	176 (12.1)	187 (11.1)	0.246
**CAD**	2456 (74.8)	1059 (72.6)	1186 (70.6)	0.005

CAD, coronary artery disease; T2DM, type 2 diabetes mellitus; TBA, total bile acid; BMI, body mass index; SBP, systolic blood pressure; DBP, diastolic blood pressure; CVD, cardiovascular disease; TC, total cholesterol; TG, triglyceride; HDL-C, high-density lipoprotein cholesterol; LDL-C, low-density lipoprotein cholesterol; ALT, alanine transaminase; AST, aspartate amino transferase; TBIL, total bilirubin; DBIL, direct bilirubin; γ-GGT, gamma-glutamyl transpeptidase; ALB, albumin; hs-TNI, hypersensitive troponin I; BNP, brain natriuretic peptide; HbA1c, glycosylated hemoglobin A1c; eGFR, estimate glomerular filtration rate; Cr, creatinine; LVEF, left ventricular ejection fraction; β-blockers, beta-blockers; ARB, angiotensin receptor blockers; ACEI, angiotensin converting enzyme inhibitors.

### Relationship Between TBA and Lipids

**Figure 2:**
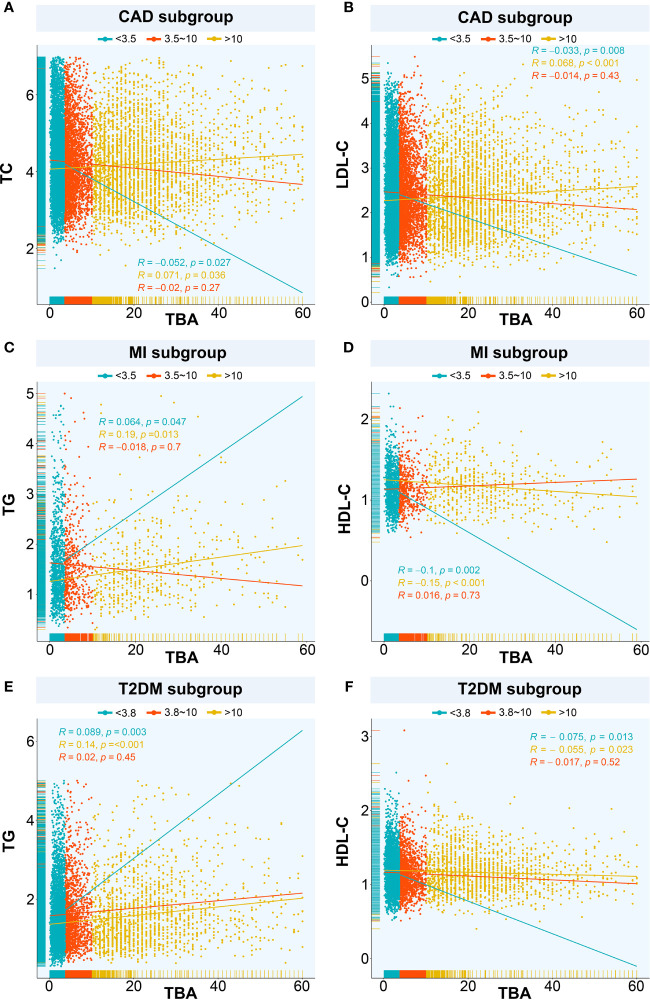
Relationships between TBA and lipids. The relationship between TBA and lipid composition in blood among the subgroups with different median TBA levels including **(A)** TBA and TC in CAD subgroup, **(B)** TBA and LDL-C in CAD subgroup, **(C)** TBA and TG in MI subgroup, **(D)** TBA and HDL-C in MI subgroup, **(E)** TBA and TG in T2DM subgroup, **(F)** TBA and HDL-C in T2DM subgroup. TBA, total bile acid; CAD, coronary artery disease; TC, total cholesterol; LDL-C, low-density lipoprotein cholesterol; TG, triglyceride; HDL-C, high-density lipoprotein cholesterol; MI, myocardial infarction; T2DM, type 2 diabetes mellitus.

Next, we explored the relationship between TBA and lipid composition in blood among the subgroups with different median TBA levels. In the CAD population (median TBA = 3.5 µmol/L), the TC level correlated negatively with low-level TBA (P < 0.05) and positively with high-level TBA (P < 0.05) (**Figure 2A**). The LDL-C level also correlated negatively with low TBA level (P = 0.008), and positively with high TBA level (P < 0.001) (**Figure 2B**). In contrary, the TG level positively correlated with both low- and high-level TBA (P < 0.001), while HDL-C negatively correlated with both low- and high-level TBA (P < 0.001). In patients with CAD combined with T2DM (median TBA = 3.5 µmol/L), the HDL-C level negatively correlated with low-level TBA (P = 0.007), while the LDL-C, TC, and TG levels positively correlated with high-level TBA (P < 0.05). In the MI population (median TBA = 3.5 µmol/L), the TG level positively correlated with low and high-level TBA (P < 0.05) (**Figure 2C**). The HDL-C level negatively correlated with low- and high-level TBA (P <0.001) (**Figure 2D**). There was no significant correlation between TBA and lipid composition in patients with MI combined with T2DM (median TBA = 3.4 µmol/L). In the T2DM subgroup (median TBA = 3.8 µmol/L), the LDL-C and TC levels correlated positively with high-level TBA (P < 0.001). TG correlated positively with low- and high-level TBA (P < 0.05) (**Figure 2E**), while HDL-C correlated negatively with low- and high-level TBA (P < 0.05) (**Figure 2F**).

### Relationship Between TBA and Disease Entities

**Figure 3:**
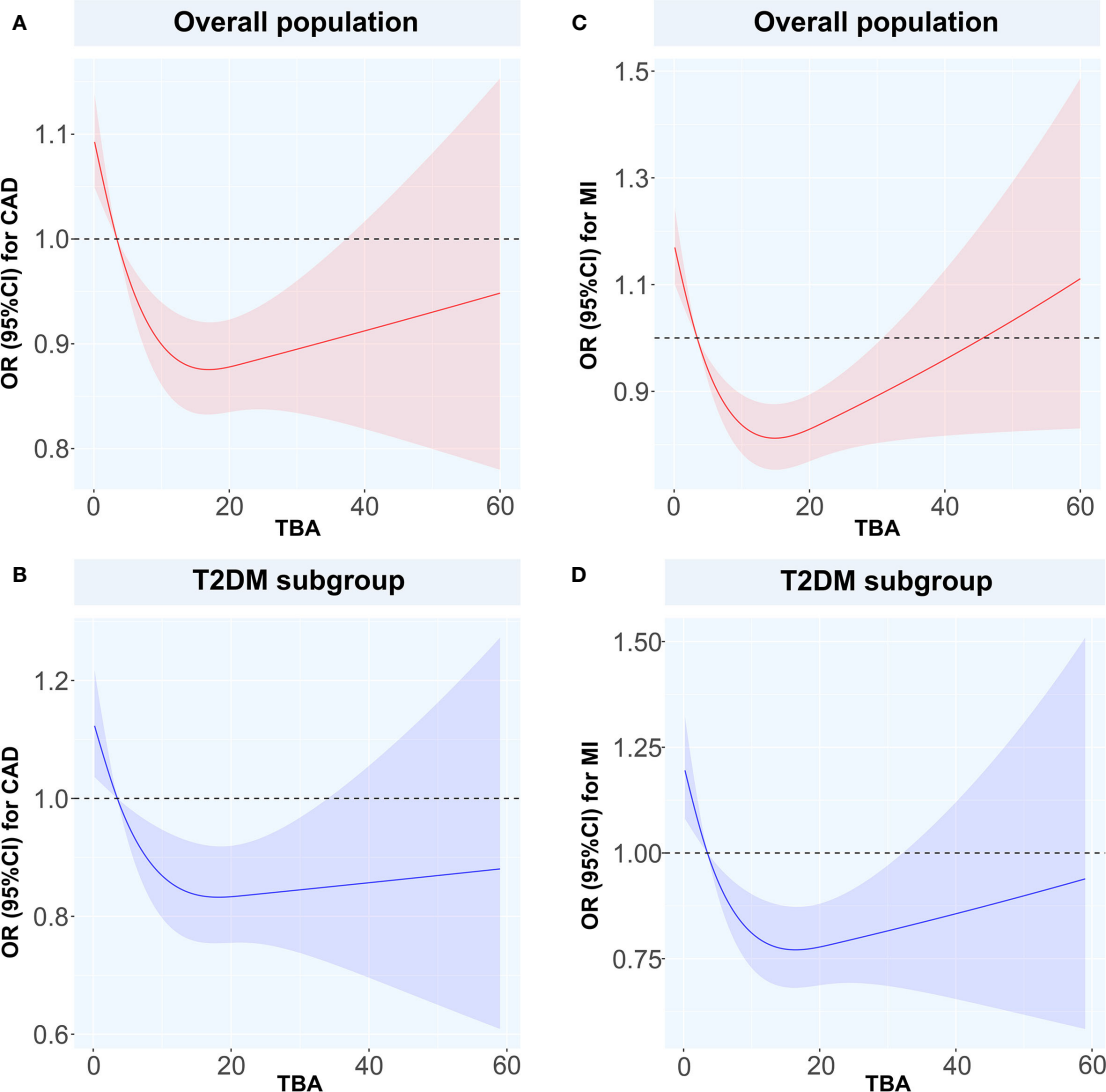
Restricted spline curves for the associations between TBA and MI or CAD Solid red and blue lines represent the odds ratio, and red and blue dashed area represent the 95% confidence intervals. **(A)** the relationship between TBA and CAD in overall population, **(B)** the relationship between TBA and CAD in T2DM subgroup, **(C)** the relationship between TBA and MI in overall population, **(D)** the relationship between TBA and MI in T2DM subgroup. TBA, total bile acid; CAD, coronary artery disease; MI, myocardial infarction; T2DM, type 2 diabetes mellitus; OR, odds ratio; CI, confidence interval.

**Figure 4:**
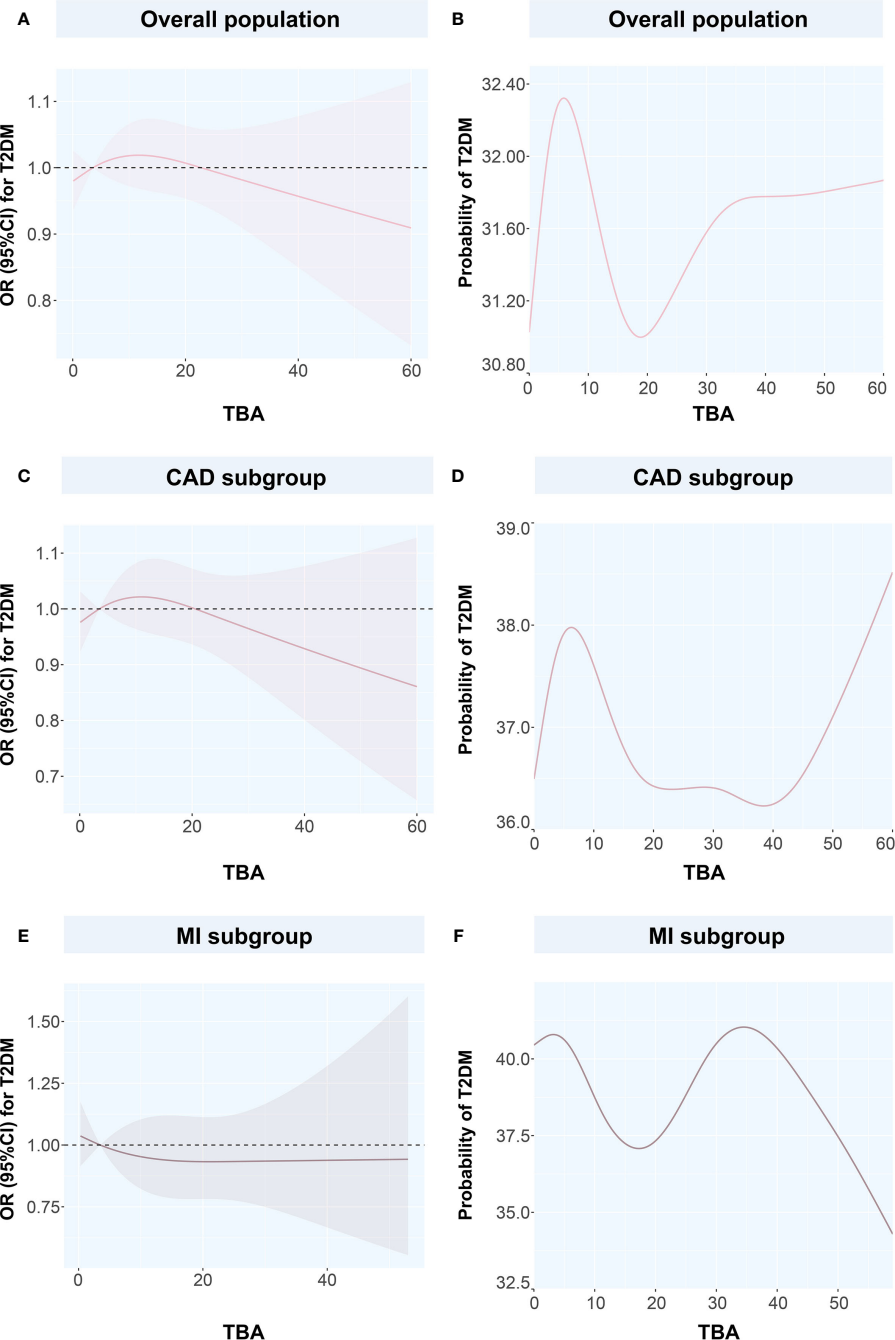
Restricted spline curves and generalized additive models for the associations between TBA and T2DM **(A)** the relationship between TBA and T2DM in overall population, **(B)** the relationship between TBA and probability of T2DM in overall population, **(C)** the relationship between TBA and T2DM in CAD subgroup, **(D)** the relationship between TBA and probability of T2DM in CAD subgroup, **(E)** the relationship between TBA and T2DM in MI subgroup, **(F)** the relationship between TBA and probability of T2DM in MI subgroup. Solid pink lines represent the odds ratio in **(A, C, E)**, while represent the probability of T2DM in **(B, D, F)**. Pink dashed area represents the 95% confidence intervals in **(A, C, E)**. TBA, total bile acid; T2DM, type 2 diabetes mellitus; CAD, coronary artery disease; OR, odds ratio; CI, confidence interval.

We used the spline analysis, with three as number of selected nodes, and after adjusted for typical influencing factors (including age, BMI, SBP, DBP and medical history of HT, hyperlipidemia, smoking, and drinking). We showed that as the level of TBA increased in CAD, MI, and CAD or MI combined with T2DM subgroups, the corresponding OR values showed an ‘L’-shaped trend curve (**Figure 3**). Furthermore, at low TBA levels, the OR values of TBA for CAD were all greater than 1. Within the normal clinical range of TBA concentrations (0–10 µmol/L), the OR value decreased as the level of TBA increased. Higher levels of TBA corresponded to OR values for CAD that showed a horizontal or slightly upward trend (**Figures 3A, B, D**) in the subgroups CAD, and CAD or MI combined with T2DM, and a large increase in the MI subgroup (**Figure 3C**). As for T2DM, in both the total and CAD populations, the OR value first showed a slight increase at first, with the increase of TBA level and then a decreasing trend (**Figures 4A, C**). In the MI subgroup, as the TBA level increased, the OR value showed a downward trend, but without reaching any significance (**Figure 4E**).

**Figure 5:**
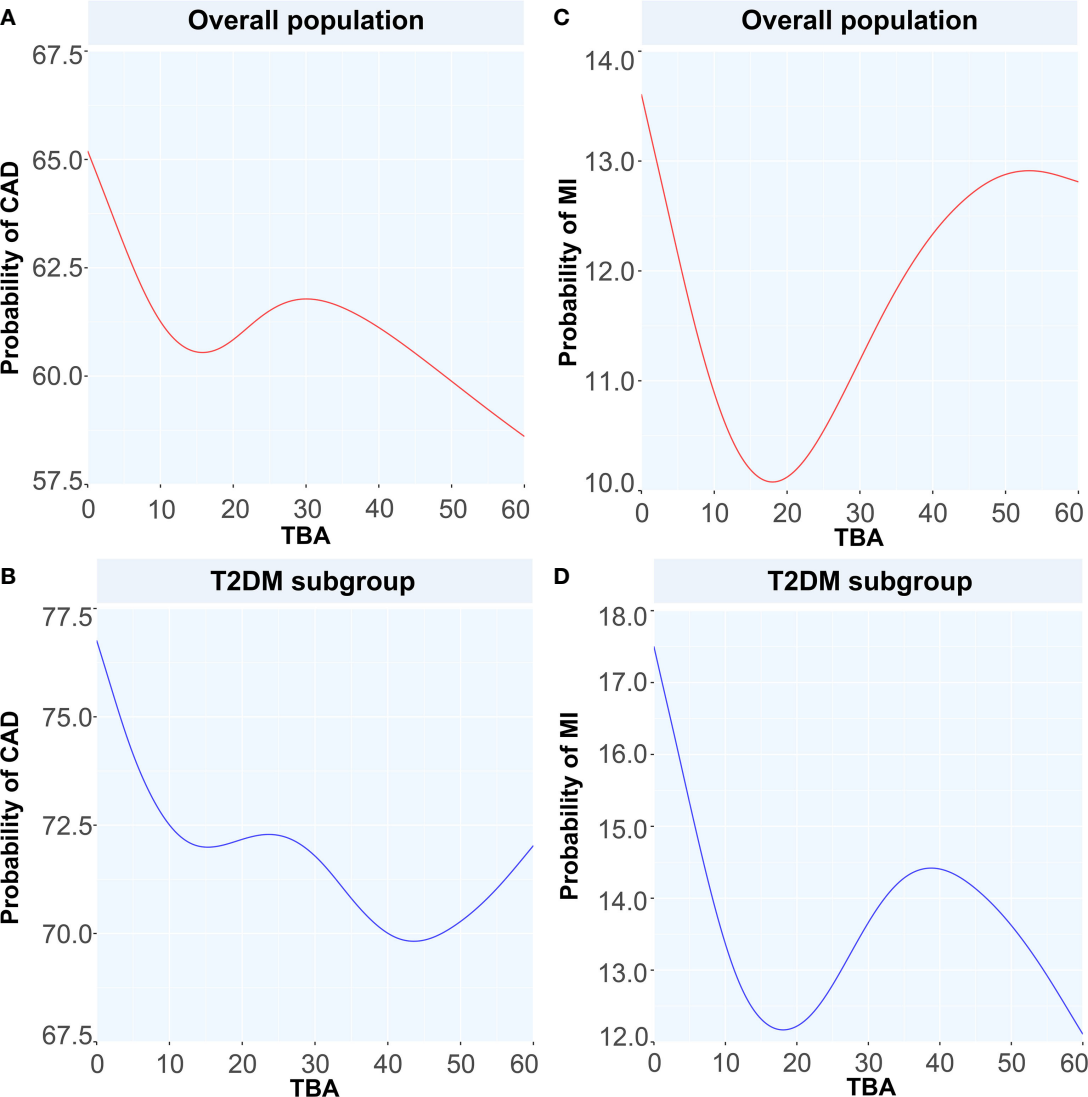
Generalized additive models for the associations between TBA and the probability of CAD and MI. Red lines represent the probability of diseases in overall population while blue lines represent the probability of diseases in T2DM subgroup. **(A)** the relationship between TBA and probability of CAD in overall population, **(B)** the relationship between TBA and probability of CAD in T2DM subgroup, **(C)** the relationship between TBA and probability of MI in overall population, **(D)** the relationship between TBA and probability of MI in T2DM subgroup. TBA, total bile acid; CAD, coronary artery disease; MI, myocardial infarction; T2DM, type 2 diabetes mellitus.

To discuss the GAM of binary variables, we used CAD, MI, T2DM, and CAD or MI combined with T2DM as the outcome variables and the TBA concentration as the predictor. The results showed that when TBA decreased, the disease state changed (**Figures 4** and **5**). The predicted probability for MI changed the most in the T2DM population, with a decrease of about 5%. However, when TBA level exceeded the normal clinical range, the characteristics of the disease varied greatly between the different populations. In the CAD population, for a TBA level higher than 10 µmol/L, the predicted probability of the disease would probably reach a peak again at 30 µmol/L (**Figure 5A**), while in the CAD population combined with T2DM, above 30 µmol/L, it is likely that the predicted probability curve conserved a U-shape (**Figure 5B**). In the MI population, the predicted probability for MI reached a peak when the TBA level reached 50–60 µmol/L (**Figure 5C**). In the MI combined with T2DM population, when the TBA level reached about 40 µmol/L, the predicted probability for MI in the T2DM population reached a peak again (**Figure 5D**). In addition, in the total and the CAD populations, the predicted probability for T2DM was substantially different from the variations observed for CVDs. At low TBA levels (approximately TBA < 5 µmol/L), the predicted probability of T2DM presented an upward trend (**Figures 4B, D**). At high concentrations of TBA (> 10 µmol/L), the predicted probability for T2DM first presented a decreasing trend and then an increasing trend. In the MI population, the predicted probability for T2DM did not vary significantly at extremely low TBA levels. When considering the range 5–15 µmol/L, the predicted probability for T2DM showed a downward trend, which transformed into an increasing trend when reaching the range of 20–35 µmol/L. At high levels of TBA (> 35 µmol/L), the predicted probability for T2DM gradually decreased (**Figure 4F**).

### Regression Analyses

Taking medium-level TBA as a reference, the univariate regression analysis indicated that in the total population, low-level TBA may be related to CAD and MI, while high-level TBA may be a protective factor against MI and T2DM. After adjusting for confounding factors, the multivariate regression analysis indicated that low-level TBA was independently associated with CAD. In contrast, high levels of TBA may be a protective factor against T2DM. Further regression analysis in the T2DM subgroup showed that higher levels of TBA may be protective against MI. After adjusting for confounding factors, including age, BMI, SBP, DBP, LDL-C and medical history of HT, hyperlipidemia, smoking, and familial CVD, the multivariate regression analysis suggested that low-level TBA was independently associated with T2DM combined with CAD. In addition, in the CAD and MI subgroups, high levels of TBA may be protective against T2DM (**Table 5**).

**Table 5:** Odds ratios of CAD, MI, T2DM and CAD/MI combined with T2DM in relation to the TBA levels.

	TBA levels	Unadjusted		Adjusted	
	μmol/L	OR (95% CI)	P value	OR (95% CI)	P value
**All population**					
**CAD**	<3.6	1.078 [1.002-1.161]	0.045	1.352 [1.192-1.534]	<0.001
	3.6~10	ref			
	>10	0.939 [0.833-1.058]	0.302	0.932 [0.801-1.084]	0.360
					
**MI**	<3.6	1.066 [0.944-1.202]	0.302	1.173 [0.945-1.455]	0.148
	3.6~10	ref		ref	
	>10	0.794 [0.650-0.970]	0.024	0.798 [0.614-1.038]	0.093
					
**T2DM**	<3.6	0.915 [0.848-0.988]	0.023	0.998 [0.876-1.137]	0.973
	3.6~10	ref		ref	
	>10	0.824 [0.727-0.935]	0.003	0.849 [0.723-0.995]	0.044
					
**Patients with T2DM**					
**CAD**	<3.6	1.135 [0.986-1.307]	0.077	1.451 [1.141-1.847]	0.002
	3.6~10	ref		ref	
	>10	0.972 [0.771-1.226]	0.811	1.005 [0.753-1.341]	0.972
					
**MI**	<3.6	1.128 [0.933-1.364]	0.213	1.102 [0.773-1.570]	0.591
	3.6~10	ref		ref	
	>10	0.679 [0.487-0.947]	0.022	0.798 [0.517-1.232]	0.309
					
**Patients with CAD**					
**MI**	<3.5	1.027 [0.907-1.161]	0.677	1.072 [0.81-1.336]	0.534
	3.5~10	ref		ref	
	>10	0.796 [0.646-0.981]	0.032	0.825 [0.630-1.082]	0.825
					
**T2DM**	<3.6	0.911 [0.830-0.999]	0.047	0.991 [0.849-1.157]	0.908
	3.6~10	ref		ref	
	>10	0.829 [0.709-0.969]	0.019	0.883 [0.726-1.073]	0.164
					
**Patients with MI**					
**T2DM**	<3.5	0.983 [0.779-1.241]	0.886	0.919 [0.599-1.410]	0.699
	3.5~10	ref		ref	
	>10	0.644 [0.430-0.964]	0.032	0.862 [0.508-1.463]	0.862

CAD, coronary artery disease; MI, myocardial infarction; T2DM, type 2 diabetes mellitus; TBA, total bile acid; OR, odds ratio; CI, confidence interval; ref, reference.

## Discussion

high-This study is the first to explore the relationship between TBA level and CAD, MI, and CAD or MI combined with T2DM in the population of menopausal women. First, this study revealed that both in the total sample and in the T2DM subgroup, TBA was significantly lower in patients with CAD. Under grouping according to the median TBA level, the percentages of CAD and MI showed a decreasing trend. Second, spline analysis indicated that with the increase of TBA level within the normal clinical range, the OR values of CAD, MI, and CAD or MI combined with T2DM showed a downward trend. Subsequently, in the GAM model and within the normal clinical range, the predicted probability for CAD, MI, and CAD or MI combined with T2DM showed a downward trend with the increase in TBA level. The logistic regression model demonstrated that low-level TBA was independently related to CAD and CAD combined with T2DM and that high-level TBA may be protective against MI and MI combined with T2DM.

### TBA and CVD

#### BAs in Lipid and Glucose Metabolism

Since BAs are only synthetized by liver cells, they can be considered as the only quantitative markers reflecting the mechanisms of cholesterol decomposition and metabolism (1). Previous studies have indicated that BA functions go beyond the sole regulation of lipid digestion and cholesterol metabolism. BAs are considered signal molecules that interact with plasma membranes and nuclear receptors. These interactions regulate the synthesis of BAs and homeostasis of energy production, as well as other important physiological processes (15). Several studies have established the important role of BAs in regulating cholesterol and TG metabolism. Moreover, the relationship between BAs and blood lipids may be bidirectional, that is, not only do BAs affect lipid and glucose metabolism or obesity, but TG metabolism also influences BA synthesis (16, 17). Interestingly, our study further supported this model for serum TBA in menopausal women. The relationship between different levels of TBA and different lipid types was variable. TC was negatively associated with low-level TBA in the CAD and CAD combined with T2DM populations, while high levels of TBA and LDL-C positively correlated in the CAD, MI, and CAD combined with T2DM populations. In addition, TG positively correlated with low- and high-level TBA in the CAD and MI populations, and high-level TBA positively correlated with TG in the CAD combined with T2DM subgroup. The relationship between other lipid components, including TC and HDL-C, and TBA may also depend on anabolic interactions between BAs and lipids to some extent.

#### Relationship Between TBA and CAD Progression

The exact relationship between the circulating TBA levels and CAD is still unclear. Some previous reports have ruled out that any significant correlation between CAD and TBA level (17). However, in recent years, an increasing number of studies on the relationship between CAD and BAs have indicated that a connection may exist. In a study based on coronary computed tomography angiography (CTA), a higher circulating TBA level was found to be an independent predictor of coronary plaque instability. The authors proposed that high TBA levels may be associated with the severity of coronary artery stenosis and risk coronary artery plaque detected by CTA (18). In another study on the role of TBA in the progression of CAD in the overall population, also using spline analysis, the presence and severity of TBA and CAD showed an L-shaped relationship, and the breakpoint was close to the upper limit of the normal TBA level (10 µmol/L). That study showed that low TBA concentration is independently and significantly related to the presence and severity of CAD, especially in presence of MI (13). In our study, both in the general population of menopausal women and in the T2DM subgroup, the average level of TBA in the CAD patients was significantly lower compared with non-CAD group. In the spline analysis, there were also certain similarities. First, within the normal clinical range (0–10 µmol/L), in menopausal women, low-level TBA is related with CAD and MI alone, or combined with T2DM. The multivariate regression analysis confirmed that low-level TBA might indeed be independently related with CAD and CAD combined with T2DM.

#### Relationship Between BA Excretion and Prognosis

As the main organic solute in bile, BAs are largely absorbed at the distal end of the small intestine, and then return to the liver where they are finally excreted, maintaining a continuous circulation of BAs between the liver and the intestine (19). A previous study found that BA excretion was significantly reduced in the CAD patients, which to a certain extent, supports the idea that CAD patients may have less BAs than non-CAD patients. Failure to effectively excrete BAs may be an independent risk factor for CAD (20). In addition, in a long-term follow-up study of BA excretion, stroke incidence, and mortality, the decrease in BAs and secondary BA excretion was related to the risk of stroke, which pointed towards an association between atherosclerosis and BA excretion as an independent risk factor for cerebrovascular disease (21). The excretion of BAs as an indicator of CAD prognosis does not have quantitative standard references, and the prognostic value of TBA level for CAD has remained unclear. Therefore, we used abundant clinical data to establish a smooth spline based on GAM. For the first time in a population of menopausal women, we uncovered the predicted probabilities for different diseases based on the changes in serum TBA levels. Within the normal clinical range (0–10 µmol/L), as the level of TBA increased, the predicted probabilities of CAD, MI, and T2DM combined with these two diseases all showed a downward trend. This was largely consistent with the results obtained by the spline analysis, but at high-level TBA (> 10 µmol/L), as the level of TBA increased, the predicted probability of disease occurrence presented variable trends. This result underlines the need for further research in exploring the underlying mechanisms.

### Various Connections Between BAs and T2DM Combined or Not With CVD

T2DM and CVDs are common clinical comorbidities, and T2DM is a recognized risk factor for CAD. Although TBA is used as a CAD marker and closely relates to metabolism, its exact relationship with T2DM and whether it affects T2DM, and thereby further affects CAD, are not clear.

#### Metabolic Characteristics of BAs Under T2DM Status

high-The abnormal composition of BAs in T2DM patients indicates that there might be an interaction between BA signal and insulin secretion capacity. In this regard, previous studies have found that fasting plasma TBA composition can be used as an indicator to predict and evaluate the progression and prognosis of T2DM (22). Moreover, changes in the circulating TBA levels are related to the pathogenesis of insulin resistance (IR) and T2DM and changing the composition of TBA may provide effective treatments for T2DM (23). A human study analyzing the changes in BA levels has revealed that compared with healthy controls, the concentration of one secondary BA, namely deoxycholic acid, was increased in T2DM patients, whereas the concentration of total BAs decreased (24). In another study, after adjusting for age and gender, the increase in the concentration of three 12α-hydroxylated secondary BA-cholic acids was associated with the occurrence of T2DM (25). Through Pearson’s test, we found that both fasting insulin level and homeostasis model assessment of IR (HOMA-IR) significantly positively correlated with serum total bile acid (P<0.05) only in the CAD population (**Supplementary Table**). Previous studies have found that IR plays an important role in regulating BA metabolism, which was not related to diabetes status (26). In addition, it has been suggested that baseline TBA levels are positively associated with T2DM risk and longitudinal changes in glucose metabolism; meanwhile, IR may partially mediate the correlation between TBA and T2DM (27). Considering the influence of obesity, which is also a risk factor for CAD, Bishay et al. have suggested that IR is closely related to incommensurate alterations to the composition of BA pool, and they have emphasized that increased BAs in IR, rather than obesity possibly contribute to the defects in insulin signaling (28). In addition, although the spline analysis failed to describe a clear relationship between TBA variation and T2DM status in menopausal women, the smooth spline analysis based on GAM indicated the potential value of TBA level to predict the probability for T2DM in the CAD and MI populations. The predicted probability for T2DM showed similar profiles in the total and CAD populations, with an initial rising trend followed by a fall according to different TBA ranges.

In addition, compared with healthy individuals, the level and composition of TBA are variable in T2DM patients, further highlighting that BA metabolism may be involved in the pathogenesis of metabolic diseases (29). In our study, in both the total sample and the T2DM subgroup of menopausal women, within different ranges of TBA level, the predicted probability for CAD varied in trend and amplitude (about 3%) with similar patterns but became inconsistent at high levels of TBA. In contrast, in the MI subgroup, TBA varied within the different ranges, and the predicted probability was about 2% lower for MI than for MI combined with T2DM. Therefore, we speculate that when T2DM is combined with CAD and MI, the body may undergo additional metabolic changes, including those in TBA and other molecules, which may affect the pathogenesis of CAD and MI combined with T2DM.

#### BA Receptors and Glucose Metabolism

Since the discovery of BA receptors, the importance of BAs as signaling molecules that regulate physiological functions has been attracting research attention. In the past 20 years, a large number of studies have revealed new functions of BAs as signaling molecules and metabolic regulators. BAs regulate signals by activating nuclear receptors such as farnesoid X receptor (FXR), and G protein-coupled receptors such as Takeda G-protein receptor 5 (TGR5) (30, 31). FXR and TGR5 agonists may play an important role in the progression of atherosclerosis and vascular calcification (32). BAs exert additional control over cholesterol metabolism by regulating many FXR target genes. Interestingly, *in vitro*, FXR activation induces the expression of low-density lipoprotein (LDL) receptors and inhibits the proprotein convertase subtilisin/kexin type 9 (PCSK9) molecule (33). In addition, the activation of BA-mediated signaling pathways may be related to enhanced control of inflammation. Previous studies have found that the TGR5 signaling pathway inhibits the activation of inflammasomes (34). FXR is involved in glucose homeostasis and lipid metabolism. Furthermore, in the intestine, BAs activate the glycogen-like peptide 1 (GLP-1) pathway through the TGR5 receptor, release insulin, stimulate resting heat generation, and reduce inflammation. Outside the intestines, BAs send signals to FXR and TGR5 receptors in the adipose tissue, skeletal muscles, and pancreas through these two receptors to maintain glucose homeostasis (35–37). This shows that at the level of molecules and signaling pathways, the TBA in plasma might act on target receptors through blood circulation and participate in inflammatory responses and glucose metabolism by activating specific pathways, thereby further influencing CVDs.

## Insight and Enlightenment

To summarize, circulating TBA levels may sometimes be a fluctuating parameter. Nevertheless, most evidence suggests that the level of circulating TBA is higher in metabolic diseases (38). In addition, previous studies have confirmed significant differences in BA levels between males and females (30). It has also been found that TBA characteristics may vary between different populations. Therefore, in this study, we focused on the specific population of menopausal women and explored the characteristics of serum TBA in CAD, MI, and CAD/MI in relation with T2DM status. This analysis provides a new reference basis to assess the relationship between clinically accessible TBA data and disease status.

## Conclusions

TBA is a common clinical biomarker that may often be ignored. As a serum marker potentially related to CAD and MI in menopausal women, TBA may be particularly valuable for patients with CAD, MI, and T2DM. As a predictor, TBA seems to present some degree of correlation with the predicted probability of disease.

## Limitations

First, our study was a single-center retrospective case-control study. Due to the large number of patients, follow-up data were unattainable. Further studies on the association between TBA and the disease are still needed in combination with the follow-up and prognosis information. Second, the TBA level in this study was based on the fasting measurement value of patients after admission. TBA data with repeated measurements were not obtained, so there may be some bias in the results.

## Data Availability Statement

The raw data supporting the conclusions of this study will be available from the corresponding author on reasonable requests.

## Ethics Statement

The studies involving human participants were reviewed and approved by the Institutional Ethics Committee of Beijing Anzhen Hospital. The data retrospectively obtained from electronic medical records.

## Author Contributions

XF and GZ made substantial contributions to manuscript writing. YZ, QG, and XF made substantial contributions to study design. XF, JY, and YL made contributions to data collection and analysis. YZ and QG revised this paper. All authors contributed to the article and approved the submitted version

## Funding

This study was supported by the grant from Natural Science Foundation of Beijing, China (Grant No. 7214223) to QG. YZ was supported by National Key Research and Development Program of China (2017YFC0908800), Beijing Municipal Health Commission (Grant No. PXM2020_026272_000002 and Grant No. PXM2020_026272_000014) and Natural Science Foundation of Beijing, China (Grant No. 7212027).

## Conflict of Interest

The authors declare that the research was conducted in the absence of any commercial or financial relationships that could be construed as a potential conflict of interest.

## Publisher’s Note

All claims expressed in this article are solely those of the authors and do not necessarily represent those of their affiliated organizations, or those of the publisher, the editors and the reviewers. Any product that may be evaluated in this article, or claim that may be made by its manufacturer, is not guaranteed or endorsed by the publisher.

## Abbreviations

BAs, Bile acids; CVDs, cardiovascular diseases; CAD, coronary artery disease; T2DM, type 2 diabetes mellitus; TBA, total bile acid; MI, myocardial infarction; LVEF, left ventricular ejection fraction; Cr, creatinine; ALT, alanine amino transferase; AST, aspartate amino transferase; γ-GGT, gamma-glutamyl transpeptidase; LDL-C, low-density lipoprotein cholesterol; TC, total cholesterol; TG, triglyceride; HDL-C, high-density lipoprotein cholesterol; TBIL, total bilirubin; DBIL, direct bilirubin; ALB, albumin; hs-TNI, hypersensitive troponin I; BNP, brain natriuretic peptide; FBG, fasting blood glucose; HbA1c, glycosylated hemoglobin A1c; RCS, restricted cube splines; GAM, generalized additive model; OR, odds ratios; CI, confidence intervals; DBP, diastolic blood pressure; BMI, body mass index; SBP, systolic blood pressure; HT, hypertension; eGFR, estimated glomerular filtration rate; ARB, angiotensin receptor blockers; ACEI, angiotensin converting enzyme inhibitor; β-blockers, beta blockers; CTA, computed tomography angiography; IR, insulin resistance; HOMA-IR, homeostasis model assessment of insulin resistance; FXR, farnesoid X receptor; TGR5, Takeda G-protein receptor 5; GLP-1, glycogen-like peptide 1.
