# Erratum: Myocardial Infarction and Coronary Artery Disease in Menopausal Women With Type 2 Diabetes Mellitus Negatively Correlate With Total Serum Bile Acids

**DOI:** 10.3389/fendo.2021.799920

**Published:** 2021-11-23

**Authors:** Frontiers Production Office

**Affiliations:** Frontiers Media SA, Lausanne, Switzerland

**Keywords:** total serum bile acids, myocardial infarction, coronary artery disease, menopausal women, type 2 diabetes mellitus

Due to a production error, “high-” was inserted in the beginning of the first paragraph of **Discussion** and sub-section **TBA and CVD**, **BAs in Lipid and Glucose Metabolism**. The correction has been implemented and the “high-” was removed from both places.

The publisher apologizes for this mistake. The original version of this article has been updated.

