# Supplementary material for: Myocardial Infarction and Coronary Artery Disease in Menopausal Women With Type 2 Diabetes Mellitus Negatively Correlate With Total Serum Bile Acids
Source: Front Endocrinol (Lausanne). 2021 Oct 5;12:754006. doi: 10.3389/fendo.2021.754006 (PMC8524089; doi:10.3389/fendo.2021.754006)
Supplement: Supplementary file 1 [file Table_1.docx]

Supplementary table. The relationship between TBA and insulin resistance

| Population | TBA and fasting insulin | | TBA and HOMA-IR | |
| --- | --- | --- | --- | --- |
|  | Pearson correlation coefficient | P value | Pearson Correlation coefficient | P value |
| All | 0.098 | 0.001 | 0.157 | < 0.001 |
| CAD | 0.101 | 0.005 | 0.076 | 0.038 |
| MI | 0.111 | 0.131 | -0.02 | 0.790 |
| T2DM | 0.075 | 0.141 | 0.033 | 0.536 |
| T2DM and CAD | 0.065 | 0.272 | 0.018 | 0.768 |
| T2DM and MI | 0.189 | 0.087 | 0.017 | 0.886 |

TBA, total bile acid; HOMA-IR, homeostasis model assessment of insulin resistance; CAD, coronary artery disease; MI, myocardial infarction; T2DM, type 2 diabetes mellitus.

HOMA-IR was calculated by the formula: HOMA-IR= fasting insulin (μU/L) x fasting glucose (nmol/L)/22.5. [1-2]

[1] Matthews DR, Hosker JP, Rudenski AS, Naylor BA, Treacher DF, Turner RC. Homeostasis model assessment: insulin resistance and beta-cell function from fasting plasma glucose and insulin concentrations in man. Diabetologia. 1985;28(7):412-419. doi:10.1007/BF00280883

[2] Shin HJ, Lee HS, Kwon YJ. Association between reproductive years and insulin resistance in middle-aged and older women: A 10-year prospective cohort study. Maturitas. 2020;142:31-37. doi:10.1016/j.maturitas.2020.07.004
